# P22 Patient-level meta-analysis of efficacy and safety from STRIVE and ReSTORE: randomized, double-blinded, multicentre Phase 2 and Phase 3 trials of rezafungin in the treatment of candidaemia and/or invasive candidiasis

**DOI:** 10.1093/jacamr/dlac133.026

**Published:** 2023-01-19

**Authors:** A Soriano, G R Thompson, O A Cornely, B J Kullberg, M Kollef, J Vazquez, P M Honore, M Bassetti, J Pullman, C Dignani, A F Das, T Sandison, P G Pappas

**Affiliations:** Hospital Clínic de Barcelona, IDIBAPS, University of Barcelona, Spain; University of California Davis Medical Center, Davis, CA, USA; University of Cologne, Faculty of Medicine and University Hospital Cologne, Department I of Internal Medicine, Excellence Center for Medical Mycology (ECMM), Cologne, Germany; University of Cologne, Faculty of Medicine and University Hospital Cologne, Chair Translational Research, Cologne Excellence Cluster on Cellular Stress Responses in Aging-Associated Diseases (CECAD), Cologne, Germany; University of Cologne, Faculty of Medicine and University Hospital Cologne, Clinical Trials Centre Cologne (ZKS Köln), Cologne, Germany; German Centre for Infection Research (DZIF), Partner Site Bonn-Cologne, Cologne, Germany; Radboud University Medical Center, Nijmegen, The Netherlands; Washington University, St Louis, MO, USA; Augusta University, Augusta, GA, USA; Brugman University Hospital, Brussels, Belgium; University of Genoa, Genoa, Italy; Mercury Street Medical, Butte, MT, USA; PSI-CRO, Durham, NC, USA; Cidara Therapeutics Inc., San Diego, CA, USA; Cidara Therapeutics Inc., San Diego, CA, USA; University of Alabama at Birmingham, Birmingham, AL, USA

## Abstract

**Background:**

Rezafungin is a next-generation echinocandin in development for treatment of candidaemia and invasive candidiasis (IC) and for prevention of invasive fungal disease caused by *Candida*, *Aspergillus* and *Pneumocystis* spp. in blood and marrow transplantation. Rezafungin once-weekly (QWk) was compared to caspofungin once-daily (QD) in two double-blind, randomized, controlled trials in patients with candidaemia and/or IC: STRIVE (Phase 2; NCT02734862) and the recently completed ReSTORE (Phase 3; NCT03667690). STRIVE demonstrated the efficacy and safety profile of rezafungin. ReSTORE showed rezafungin noninferiority to caspofungin for 30 day all-cause mortality (ACM) and global response at Day 14 with comparable safety. Patient-level meta-analyses of efficacy and safety from both trials are presented.

**Methods:**

Details of STRIVE and ReSTORE were previously described. In this analysis of data from both trials, patients who received rezafungin QWk (400 mg in Week 1, then 200 mg) were compared with those who received caspofungin QD (70 mg on Day 1 followed by 50 mg) for ≥14 days (up to 4 weeks). Efficacy endpoints included 30 day ACM (primary US FDA), mycological response at Day 5 (secondary), and time to first negative blood culture (TTNBC) (exploratory). Safety was evaluated by adverse events (AEs).

**Results:**

Groups were well matched (Table 1). Figure 1 shows 30 day ACM (overall and by final diagnosis). Mycological response at Day 5 was 73.4% (102/139) and 64.5% (100/155) in rezafungin and caspofungin groups, respectively (difference=9.5, 95% CI=−0.9, 19.9). In patients with positive blood culture before randomization, median TTNBC was 22.3 h in rezafungin-treated versus 26.3 h in caspofungin-treated patients (stratified log rank p=0.0034, not adjusted for multiplicity). The summary of AEs (Table 2) demonstrates similar outcomes for rezafungin and caspofungin groups.

**Conclusions:**

In the Phase 2/3 patient-level meta-analysis, rezafungin QWk demonstrated efficacy with a similar 30 day ACM rate and safety comparable to that of caspofungin QD. Data for mycological eradication at Day 5 and TTNBC support results from the primary efficacy endpoint and provide initial evidence for the theory that high, front-loaded drug exposure leads to faster fungal clearance. Further analysis of this integrated dataset may provide additional insights on rezafungin efficacy and safety.

**Table 1.** Baseline demographics/characteristics in the patient-level meta-analysis of STRIVE and ReSTORE (mITT population^a^)

Demographic or characteristic	Rezafungin 400 mg/200 mg weekly (*N*=139)	Caspofungin 70 mg/50 mg daily (*N*=155)
Age, years, mean ± SD (range)	59.8 ± 15.7 (19, 91)	60.8 ± 15.0 (20, 93)
Age <65 years, *n* (%)	82 (59.0)	92 (59.4)
Age ≥65 years, *n* (%)	57 (41.0)	63 (40.6)
Female, *n* (%)	49 (35.3)	65 (41.9)
Race, *n* (%)		
ȃAsian	24 (17.3)	34 (21.9)
ȃBlack or African American	11 (7.9)	8 (5.2)
ȃWhite	95 (68.3)	106 (68.4)
ȃOther/not reported	9 (6.5)	7 (4.5)
Final diagnosis, *n* (%)		
ȃCandidaemia	100 (71.9)	115 (74.2)
ȃInvasive candidiasis^b^	39 (28.1)	40 (25.8)
Modified APACHE II score, *n* (%)^c^		
ȃ≥20	21 (15.1)	26 (16.8)
ȃ<20	116 (83.5)	126 (81.3)

mITT, modified ITT.

mITT population: patients with positive cultures from blood or another normally sterile site ≤96 h before randomization.

IC group includes some subjects with deep tissue infection and candidaemia.

Reported for patients with APACHE II score data available.

**Figure 1.**
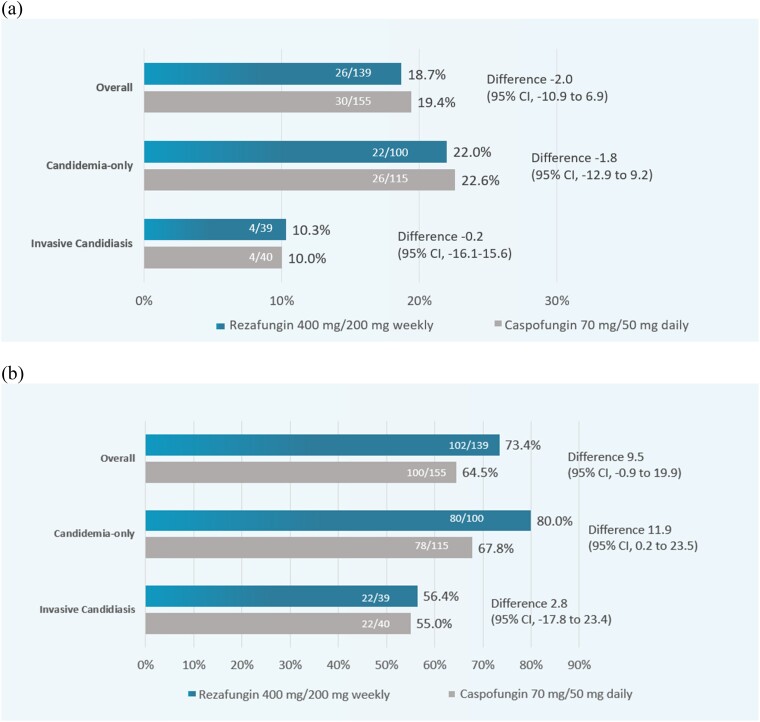
Primary and secondary efficacy endpoints in the patient-level meta-analysis of STRIVE and ReSTORE: overall and by final diagnosis (mITT population). Data could not be combined for the primary endpoints of global cure at Day 14 (ReSTORE) and overall cure at Day 14 (STRIVE) due to differences in the definitions and methods of each study. (a) 30 day ACM. (b) Mycological eradication on Day 5.

**Table 2.** Summary of TEAE data from the patient-level meta-analysis of STRIVE and ReSTORE (safety population)

Parameter	Number of subjects (%)	
	Rezafungin 400 mg/200 mg weekly (*N*=151)	Caspofungin 70 mg/50 mg daily (*N*=166)
≥1 TEAE	138 (91.4)	138 (83.1)
Study drug-related TEAE	22 (14.6)	18 (10.8)
Serious TEAE	83 (55.0)	81 (48.8)
TEAE leading to study drug discontinuation	14 (9.3)	15 (9.0)

TEAE, treatment-emergent adverse event.

